# The actual implementation status of self-isolation among Japanese workers during the COVID-19 outbreak

**DOI:** 10.1186/s41182-020-00250-7

**Published:** 2020-08-03

**Authors:** Masaki Machida, Itaru Nakamura, Reiko Saito, Tomoki Nakaya, Tomoya Hanibuchi, Tomoko Takamiya, Yuko Odagiri, Noritoshi Fukushima, Hiroyuki Kikuchi, Shiho Amagasa, Takako Kojima, Hidehiro Watanabe, Shigeru Inoue

**Affiliations:** 1 Department of Preventive Medicine and Public Health, Tokyo Medical University, 6-1-1 Shinjuku, Shinjuku-ku, Tokyo, 160-8402 Japan; 2 Department of Infection Prevention and Control, Tokyo Medical University Hospital, 6-7-1 Nishishinjuku, Shinjuku-ku, Tokyo, 160-0023 Japan; 3 Division of International Health (Public Health), Graduate School of Medical and Dental Sciences, Niigata University, 1-757, Asahimachi-dori, Niigata, Niigata 951-8510 Japan; 4 Graduate School of Environmental Studies, Tohoku University, Aoba, 468-1, Aramaki, Aoba-ku, Sendai, Miyagi 980-0845 Japan; 5 Department of International Medical Communications, Tokyo Medical University, 6-7-1 Nishishinjuku, Shinjuku-ku, Tokyo, 160-0023 Japan

**Keywords:** COVID-19, viral infection, pandemic, outbreak, self-isolation, protective measures, epidemiology, public health

## Abstract

**Background:**

Self-isolation is an important personal protective measure in inhibiting the transmission of coronavirus disease 2019 (COVID-19) as people carry out economic and social activities amid its spread. Yet few studies have clarified the actual implementation status of self-isolation during an outbreak. This study aimed to reveal the actual implementation of self-isolation among Japanese workers during the COVID-19 outbreak and the factors inhibiting this measure.

**Methods:**

This was a cross-sectional study based on an internet survey completed by 1,226 workers (60.0% men) living in 7 prefectures (i.e., Tokyo, Kanagawa, Saitama, Chiba, Ibaraki, Tochigi, and Gunma) who were selected among registrants of an Internet research company, between May 12 and 17, 2020. Participants were asked whether they had experienced fever or other cold symptoms between February 17, 2020 and the date of the survey. Those who responded affirmatively were asked where they had visited (e.g., hospital, work, and shopping for groceries or necessities) to clarify whether they had left the house within 7 days after symptom onset. We performed multivariate logistic regression analysis to clarify the relationship between going to work within 7 days after symptom onset and both sociodemographic factors and employment-related constraints.

**Results:**

Of the survey participants, 82 had experienced fever or other cold symptoms (6.7%). Among these participants, 51 (62.2%) went to work within 7 days after symptom onset. A mere 17.1% practiced strict self-isolation. Multivariate logistic regression analysis revealed that those living outside the metropolitan area (i.e., Ibaraki, Tochigi, and Gunma), working as a company employee, and being unable to work from home were associated with going to work within 7 days after symptom onset.

**Conclusions:**

The prevalence of strict self-isolation among participants who experienced cold-like symptoms during the COVID-19 outbreak was extremely low, and 62.2% of these participants went to work within 7 days after symptom onset. This study highlights the need for further public awareness regarding self-isolation and countermeasures against factors that obstruct it.

## Background

As of May 2020, the coronavirus disease 2019 (COVID-19) pandemic remains active. It is vital that personal protective measures are implemented by the public as a method to mitigate the epidemic of respiratory viruses such as COVID-19, especially before a well-matched vaccine is widely available [1]. One personal protective measure recommended by the World Health Organization in the COVID-19 pandemic is self-isolation [2]. Self-isolation refers to voluntary separation or restriction of movement of ill persons in a designated room to prevent transmission to others in their own homes or elsewhere [3]. This method is effective in preventing individuals who have been infected from transmitting the virus to others [1]. Many health authorities are recommending self-isolation during the COVID-19 pandemic [2, 4, 5]. This includes the Japanese Ministry of Health, Labour and Welfare that has been recommending the public practice of self-isolation, if a person develops a fever or other cold symptoms, since the earliest time of the outbreak [6]. Most research on the practice of self-isolation by ordinary citizens have evaluated the intention to practice self-isolation and have reported the prevalence of self-isolation to be approximately 70-90% [7–10]. In contrast, there are very few studies evaluating the actual status of self-isolation [11]. Furthermore, previous studies have found employment characteristics, such as being unable to work from home, no guaranteed paid leave, and low income as factors inhibiting the practice of self-isolation [9, 12], however to the best of our knowledge, none have explored factors obstructing self-isolation while looking at the actual status of self-isolation practices. As of May 2020, many countries including Japan are beginning to relax restrictions placed on outings by various state of emergency declarations [13, 14]. Self-isolation by ordinary citizens will be an important personal protective measure for public health as we resume social activities [15]. Furthermore, self-isolation by workers will be of great importance, since there is a possibility that employment-related constraints which cannot be controlled by workers themselves may have a major impact on the implementation of self-isolation, and workers are likely to have more contact with co-workers due to long working hours.

Thus, this study aimed to clarify the actual implementation of self-isolation among Japanese workers who experienced fever or other cold symptoms during the COVID-19 outbreak and factors inhibiting this measure.

## Methods

### Study sample and data collection

This was a cross-sectional study utilizing data of the third wave of a longitudinal research which aimed to clarify implementing personal protective measures by ordinary citizens during the COVID-19 outbreak. In the first wave survey of this longitudinal study on February 25, 2020, a total of 2,400 men and women aged 20 to 79 years (sampling by sex and 10-year age groups; 12 groups, n=200 in each group) who were living in 7 prefectures (i.e., Tokyo, Kanagawa, Saitama, Chiba, Ibaraki, Tochigi, and Gunma), and who met the criteria to participate in this research were recruited from 8,156 registrants, of a Japanese Internet research service company called MyVoice Communication, Inc., which had approximately 1.12 million registered participants as of January 2020. Detailed methods about sampling have been reported elsewhere [10]. The company reached out to these 2400 potential respondents by e-mail, to participate in the third wave research on May 12. The questionnaires were placed in a secured section of a website, and potential respondents received a specific URL in their e-mail. Participation was voluntary, and participants responded to the questionnaire by accessing the specified URL. The response cutoff date was May 17. Reward points valued at 50 yen were provided as incentives for participation (approximately 0.5 US dollars as of May 2020).

### Assessment of actual implementation of self-isolation

The Japanese Ministry of Health, Labour and Welfare has recommended that ordinary citizens practice self-isolation if they have fever or other cold symptoms [6]. Therefore, the present study evaluated the self-isolation status of individuals who experienced fever or other cold symptoms regardless of whether COVID-19 was diagnosed (in this study, such symptoms and participants are referred to as having ‘cold-like symptoms’ and ‘participants who have experienced cold-like symptoms’, respectively). The recommended self-isolation period was for 7 days after symptom onset according to guidelines of the Japanese Society of Travel and Health and the April 4 Interim Guidance from the US Centers for Disease Control and Prevention [16, 17].

Participants were first asked, “Have you experienced fever or other cold symptoms since February 17, 2020?” The date February 17, 2020, was when the Japanese Ministry of Health, Labour and Welfare requested citizens to begin practicing self-isolation [6]. Participants who responded “yes” were then asked whether they had gone out to various places (e.g., hospital, work, shopping for groceries or necessities) within 7 days of symptom onset, and responded with either a “yes” or “no”).

### Assessment of sociodemographic factors and employment-related constraints

In the first wave survey, participants stated their sex, age, underlying diseases which included heart diseases, respiratory diseases, kidney diseases, diabetes, and hypertension (yes/no), and residential area (metropolitan area [i.e., Tokyo, Kanagawa, Saitama, and Chiba]/nonmetropolitan area [i.e., Ibaraki, Tochigi, and Gunma]). In the third wave survey, participants stated their occupation (company employee, self-employed, part-time job, government worker, homemaker, unemployed, student, other) and responded to the following about employment-related constraints that may inhibit the practice of self-isolation: can work from home (yes/no), will not be paid if leave is taken (yes/no), and may be terminated if leave is taken (yes/no) [12]. The research company also provided categorized data as follows: living arrangement (with others/alone), educational attainment (university graduate level or above) and household income level (< 5 million yen or ≥ 5 million yen).

### Statistical analysis

As this study aimed to reveal the actual implementation status of self-isolation practices among workers, statistical analyses included only subjects who selected ‘company employee’, ‘self-employed’, ‘part-time job’, ‘government worker’, or ‘other’ for their occupation. Participants who had experienced cold-like symptoms and responded that they had not gone anywhere or had gone only to the hospital within 7 days after symptom onset were classified as practicing ‘strict self-isolation’, while those who went out for reasons other than hospital visits were classified as ‘not practicing strict self-isolation’. The total number of persons and percentages were calculated for each of these groups, as well as for each location mentioned. Participant characteristics were compared using the chi-square test, Fisher’s exact test, or t-test between participants who did or did not experience cold-like symptoms, and among those who experienced cold-like symptoms, whether or not they did or did not go to work within 7 days after symptom onset.

To clarify the association between going to work within 7 days of symptom onset and each sociodemographic factor and employment-related constraints, a multivariate logistic regression analysis was performed. The participants of this logistic regression analysis were those who responded that they experienced fever or other cold symptoms since February 17, 2020. The dependent variable was set as a dichotomous variable coded as “1” if the participant who experienced fever or other cold symptoms went to work within 7 days of symptom onset, and “zero” otherwise. The independent variables included sex, age (≥ 65 years old/< 65 years old), underlying diseases (yes/no), residential area (metropolitan area/nonmetropolitan area), living arrangement (with others/alone), educational attainment (university graduate level or above/below), household income level (< 5 million yen/≥ 5 million yen), occupation (company employee/self-employed/part-time job/ government worker, or other), ability to work from home (yes/no), absence of paid leave (yes/no), and possibility of termination if leave is taken (yes/no).

Statistical analyses were performed using IBM SPSS Statistics for Windows, version 26 (IBM Japan, Tokyo, Japan). Two-sided *p* values less than 0.05 were considered to be statistically significant.

## Results

The internet research company reached out to 2385 participants, after excluding participants who were not registered with the company at the time of the third wave survey (n=15), and 2,200 participants responded to the questionnaire. Of the 2,200 potential respondents, 974 were excluded for the following reasons: they were homemakers, unemployed, or students (n=810), and the data provided by the research company were incomplete (n=164). Therefore, the analysis set consisted of 1,226 participants.

Of the analysis set, 82 participants (6.7%) experienced cold-like symptoms between February 17, 2020, and the day of the survey. No participant reported that they were diagnosed with COVID-19. Table 1 shows the participant characteristics. Compared to participants who did not experience cold-like symptoms, the proportion of participants having underlying diseases and not being paid if leave is taken was significantly higher among participants who experienced cold-like symptoms.

**Table 1:** Participant characteristics

	Total		Did not experience cold-like symptoms ^a^		Experienced cold-like symptoms		
	n=1,226 (100%)		n=1,144 (93.3%)		n=82 (6.7%)		
	n (%)/mean (SD)		n (%)/mean (SD)		n (%)/mean (SD)		p-value
Sex (men)	735	60.0%	690	60.3%	45	54.9%	0.332^d^
Age, years	46.3	13.5	46.4	13.5	45.5	13.3	0.517^e^
Underlying diseases ^b^ (yes)	264	21.5%	239	20.9%	25	30.5%	0.041^d^
Residential area (metropolitan area ^c^)	1,120	91.4%	1,048	91.6%	72	87.8%	0.236^d^
Living arrangement (with others)	938	76.5%	872	76.2%	66	80.5%	0.379^d^
Educational attainment (university graduate level or above)	744	60.7%	698	61.0%	46	56.1%	0.379^d^
Household income level (≥ 5 million yen)	768	62.6%	718	62.8%	50	61.0%	0.747^d^
Occupation							
Company employee	761	62.1%	716	62.6%	45	54.9%	0.095^d^
Self-employed	107	8.7%	97	8.5%	10	12.2%	
Part-time job	247	20.1%	224	19.6%	23	28.0%	
Government worker, Other	111	9.1%	107	9.4%	4	4.9%	
Employment-related constraints that may obstruct self-isolation practices							
Can work from home (no)	640	52.2%	592	51.7%	48	58.5%	0.235^d^
Will not be paid if leave is taken (yes)	570	46.5%	523	45.7%	47	57.3%	0.042^d^
May be terminated if leave is taken (yes)	284	23.2%	263	23.0%	21	25.6%	0.587^d^

SD, standard deviation

a Fever or other cold symptoms

b Underlying diseases included heart diseases, respiratory diseases, kidney diseases, diabetes, and hypertension.

c Included Tokyo, Kanagawa, Saitama, and Chiba prefecture

p-value was calculated using ^d^ chi-square test or ^e^ t test, as appropriate

Table 2 shows the behavior of participants who experienced cold-like symptoms within 7 days of symptom onset amid the COVID-19 outbreak. Of the 82 participants who experienced cold-like symptoms, a mere 17.1% practiced strict self-isolation for the 7-day period after onset. The most common locations for outings among participants who did not practice self-isolation were the workplace and shops for groceries or necessities with 51 (62.2%) of these participants going to work within 7 days after symptom onset.

**Table 2:** Behavior of participants who experienced cold-like symptoms within 7 days of symptom onset amid COVID-19 outbreak

Total number of participants who experienced cold-like symptoms ^a^	n=82	
Status of implementation of strict self-isolation		
	n (%)	
Implementing strict self-isolation	14	17.1%
Did not go out	9	11.0%
Went out for hospital visits only	5	6.1%
Did not practice strict self-isolation		
Went out for reasons other than hospital visits	68	82.9%
Percentage of participants who went out to each location within 7 days after symptom onset		
	n (%)	
Workplace	51	62.2%
Shops for groceries or necessities	58	70.7%
Event or gathering	5	6.1%
Lessons (for leisure)	5	6.1%
Restaurants	17	20.7%
Other places for different reasons	24	29.3%

^a^Participants who responded that they had experienced cold-like symptoms (fever or other cold symptoms) between February 17, 2020, and the day of the survey (May 12 to May 17)

Table 3 shows the characteristics of participants who experienced cold-like symptoms categorized by whether the patient did or did not go to work within 7 days after symptom onset. Among participants who experienced cold-like symptoms and went to work within 7 days after symptom onset, although the differences were not statistically significant, the proportion of participants who were unable to work from home was higher than participants who were able to do so.

**Table 3:** Characteristics of patients who experienced cold-like symptoms categorized by whether they went to work within 7 days after symptom onset (n=82)

	Participants who experienced cold-like symptoms	Went to work within 7 days after symptom onset		
	n	n	%	p-value
All	82	51	62.2%	
Sex:				
Men	45	26	57.8%	0.363^c^
Women	37	25	67.6%	
Age, years				
Older adults	8	4	50.0%	0.454^c^
Under 65 years	74	47	63.5%	
Underlying diseases ^a^:				
Yes	25	12	48.0%	0.079^c^
No	57	39	68.4%	
Residential area:				
Metropolitan area ^b^	72	43	59.7%	0.215^c^
Nonmetropolitan area ^b^	10	8	80.0%	
Living arrangement:				
With others	66	39	59.1%	0.239^c^
Alone	16	12	75.0%	
Educational attainment:				
University graduate level or above	46	30	65.2%	0.523^c^
Below	36	21	58.3%	
Household income:				
≥ 5 million yen	50	30	60.0%	0.608^c^
< 5 million yen	32	21	65.6%	
Occupation				
Company employee	45	33	73.3%	0.054^d^
Self-employed	10	3	30.0%	
Part-time job	23	13	56.5%	
Government worker, Other	4	2	50.0%	
Employment-related constraints that may obstruct self-isolation practices				
Can work from home:				
Yes	34	18	52.9%	0.146^c^
No	48	33	68.8%	
Will not be paid if leave is taken:				
Yes	47	28	59.6%	0.571^c^
No	35	23	65.7%	
May be terminated if leave is taken:				
Yes	21	13	61.9%	0.975^c^
No	61	38	62.3%	

^a^Underlying diseases included heart diseases, respiratory diseases, kidney diseases, diabetes, and hypertension

^b^Included Tokyo, Kanagawa, Saitama, and Chiba prefecture

p-value was calculated using ^c^ chi-square test or ^d^ Fisher’s exact test, as appropriate

Table 4 shows the association between going to work within 7 days after symptom onset and each sociodemographic factor and employment-related constraints among participants who experienced fever or other cold symptoms (n=82). According to multivariate logistic regression analysis, being a company employee, and being unable to work from home were significant factors for those who went to work within 7 days after symptom onset (being a company employee; Odds ratio [OR]: 25.81, 95% confidence interval [95% CI]: 2.23-298.31, unable to work from home; OR: 4.22, 95% CI: 1.02-17.43), while living in a metropolitan area was a significant factor for not going to work (living in a metropolitan area; OR: 0.06, 95% CI: 0.00-0.79).

**Table 4:** The association between going to work within 7 days after symptom onset and each sociodemographic factor and employment-related constraints among participants who experienced cold-like symptoms (n=82)

	n	Odds ratio ^a^ (95% confidence interval)
Sociodemographic factor		
Sex:		
Men	45	Ref
Women	37	1.09 (0.32-3.75)
Age:		
Older adults	8	0.58 (0.10-3.30)
Under 65 years	74	Ref
Underlying diseases ^b^:		
Yes	25	Ref
No	57	2.37 (0.69-8.09)
Residential area:		
Metropolitan area ^c^	72	0.06 (0.00–0.79) *
Nonmetropolitan area ^c^	10	Ref
Living arrangement:		
With others	66	0.41 (0.07-2.52)
Alone	16	Ref
Educational attainment:		
University graduate level or above	46	0.98 (0.19–5.03)
Below	36	Ref
Household income:		
≥ 5 million yen	50	1.17 (0.32–4.30)
< 5 million yen	32	Ref
Occupation		
Company employee	45	25.81 (2.23-298.31) *
Self-employed	10	Ref
Part-time job	23	5.62 (0.44–71.97)
Government worker, Other	4	5.16 (0.18–148.50)
Employment-related constraints that may obstruct self-isolation practices		
Can work from home:		
Yes	34	Ref
No	48	4.22 (1.02–17.43) *
Will not be paid if leave is taken:		
Yes	47	1.12 (0.19–6.47)
No	35	Ref
May be terminated if leave is taken:		
Yes	21	0.72 (0.16–3.27)
No	61	Ref

*p-value: <0.05

^a^Odds ratios were calculated and adjusted for all individual variables

^b^Underlying diseases included heart diseases, respiratory diseases, kidney diseases, diabetes, and hypertension

^c^Metropolitan area included Tokyo, Kanagawa, Saitama, and Chiba prefecture, and nonmetropolitan area included Ibaraki, Tochigi, and Gunma

## Discussion

We set out to determine the actual implementation status of self-isolation among Japanese workers during the COVID-19 outbreak and to clarify the factors that may inhibit the implementation of self-isolation. Only 17.1% of participants who experienced cold-like symptoms practiced strict self-isolation within 7 days after symptom onset, while 62.2% went to work during this period. Multivariate logistic regression analysis indicated that being unable to work from home, living outside a metropolitan area, and being a company employee may be factors associated with people going to work during the recommended self-isolation period.

The majority of previous research on self-isolation have evaluated the intention to practice self-isolation and results have shown that the prevalence of self-isolation by ordinary citizens was approximately 70-90% [7–10]. Conversely, Nonaka et al. found that during the 2009 pandemic of the novel swine-origin influenza A in Japan, 13 of 14 patients (93%) who were given a diagnosis of influenza at a hospital, visited their workplace, school, or other potentially crowded settings within 7 days after diagnosis [11]. This is consistent with the findings of the present study in which the prevalence of strict self-isolation was extremely low in the majority of participants who experienced cold-like symptoms as many of them went out to work or shop within 7 days after symptom onset. Previous researches evaluating the intent to self-isolate may be overestimating the prevalence of self-isolation due to social desirability bias, in which respondents answered in such a way that they would be viewed favorably [18]. Our results suggest that most Japanese workers do not practice strict self-isolation during a pandemic. Further public awareness activities concerning the importance of self-isolation and promoting commitment to its practice are essential.

The employment-related constraint of being unable to work from home and the sociodemographic factors of living outside a metropolitan area, and being a company employee were associated with participants going to work during the recommended self-isolation period in the present study. In two studies of self-isolation conducted in the United States, the employment characteristics of being unable to work from home and not being paid while on leave, as well as having a low income, were found to be factors impeding self-isolation [7, 12]. A separate study by Bodas et al. concerning intention to self-isolate during the COVID-19 outbreak in Israeli adults reported that guaranteed income during self-isolation increased the intention to practice self-isolation [9]. Thus, our finding that being unable to work from home was a factor obstructing self-isolation is indeed consistent with these previous studies, however in contrast, we did not find statistically significant associations between going to work within 7 days after symptom onset and guaranteed income while on leave, or relation with income levels. As the sample of participants for the multivariate analysis in this study was small (n=82), we would like to be reserved when drawing conclusions, yet it seems likely that the impact of employment characteristics and sociodemographic factors on self-isolation practices differs depending on cultural and ethnic backgrounds. There is a tendency in Japanese culture to portray a responsible worker as someone who actually goes into the workplace to work. It is further probable that if workers were given the option of working from home when they are feeling unwell, the practice of self-isolation would increase voluntarily, thereby preventing the spread of infection.

Aside from work, participants also commonly went out during the recommended self-isolation period to shop for groceries and necessities. During the current COVID-19 pandemic, the European Centre for Disease Prevention and Control recommends that individuals practicing self-isolation call on a friend, neighbor, or health-care worker to purchase groceries and the like on their behalf [19]. Such efforts are also likely to improve the status of self-isolation practices.

There are some limitations that should be considered in our study. The most important point is the fact that in this study, participants were recruited from people enrolled in a single Internet research company, and thus, the results may have been affected by selection bias. Relatively little is known about the characteristics of people in online communities [20]. Furthermore, some sociodemographic factors (e.g. age, sex and residential area) of the participants in this study were different from that of the Japanese population [21], since the sociodemographic factor of the research company registrant differs from that of the general Japanese population. Second, this study has a small sample of participants who experienced cold-like symptoms (n=82). The number of independent variables may be slightly large considering the sample size [22]. A larger-scale survey will be necessary to further clarify the factors obstructing actual self-isolation practices. Third, concerning the actual status of self-isolation practices, our survey asked participants about symptom onset between February 17, 2020, and the day of the survey, however there were major changes in the spread of COVID-19 during this time. As self-restraint requests by the Japanese government also escalated during this time [23, 24], these changes may have influenced the status of self-isolation practices by workers. As we did not ask the participants about the date of onset, the influence of such changes is unclear. Fourth, since we used the questionnaire to assess symptom onset and behavior within 7 days after symptom onset, these results may be affected by recall bias [25]. Fifth, the participants in this study participated in a longitudinal research which aimed to clarify implementing personal protective measures by ordinary citizens during the COVID-19 outbreak from February 25, 2020. Participation in this longitudinal study may have influenced the participants’ awareness of personal protective measures, thereby possibly affecting the implementation of self-isolation. Finally, the results, especially the prevalence of self-isolation, may only be directly applied to Japanese populations. In the case of other populations with different cultural, ethnic, and geographical backgrounds, the prevalence of personal protective measures may vary greatly when compared with those reported in the present survey. Despite these limitations, to the best of our knowledge, this study is the first to reveal the actual status of self-isolation practices of workers during the COVID-19 outbreak.

## Conclusion

The prevalence of strict self-isolation among participants who experienced cold-like symptoms during the COVID-19 outbreak was extremely low, and 62.2% of these participants went to work within 7 days after symptom onset. Providing workers with the option to work from home may be an effective means to increase the prevalence of self-isolation. This study emphasizes the need for further public awareness regarding self-isolation and measures to counter the factors that impede this important measure to prevent transmission of COVID-19.

## Data Availability

The datasets used and/or analyzed during the current study are available from the corresponding author on reasonable request.

## Abbreviations

COVID-19: coronavirus disease 2019

## References

[1] Qualls N, Levitt A, Kanade N, Wright-Jegede N, Dopson S, Biggerstaff M (2017). Community mitigation guidelines to prevent pandemic influenza - United States, 2017. MMWR Recomm Rep..

[2] World Health Organization. Protecting yourself and others from the spread COVID-19. 2020. https://www.who.int/emergencies/diseases/novel-coronavirus-2019/advice-for-public. Accessed 4 June 2020.

[3] European Centre for Disease Prevention and Control. Guide to public health measures to reduce the impact of influenza pandemics in Europe – ‘The ECDC Menu’. 2009. https://www.ecdc.europa.eu/sites/default/files/media/en/publications/Publications/0906_TER_Public_Health_Measures_for_Influenza_Pandemics.pdf. Accessed 4 June 2020.

[4] European Centre for Disease Prevention and Control. Guidelines for the use of non-pharmaceutical measures to delay and mitigate the impact of 2019-nCoV. 2020. https://www.ecdc.europa.eu/en/publications-data/guidelines-use-non-pharmaceutical-measures-delay-and-mitigate-impact-2019-ncov. Accessed 4 June 2020.

[5] U.S. Centers for Disease Control and Prevention. What to do if you are sick. 2020. https://www.cdc.gov/coronavirus/2019-ncov/if-you-are-sick/steps-when-sick.html. Accessed 4 June 2020.

[6] Japanese Ministry of Health, Labour and Welfare. Prevention measures against coronavirus disease 2019 (COVID-19). 2020. https://www.mhlw.go.jp/content/10900000/000607599.pdf. Accessed 4 June 2020.

[7] Blendon RJ, Koonin LM, Benson JM, Cetron MS, Pollard WE, Mitchell EW (2008). Public response to community mitigation measures for pandemic influenza. Emerg Infect Dis..

[8] Brown LH, Aitken P, Leggat PA, Speare R (2010). Self-reported anticipated compliance with physician advice to stay home during pandemic (H1N1) 2009: results from the 2009 Queensland Social Survey. BMC Public Health..

[9] Bodas M, Peleg K. Self-isolation compliance in the COVID-19 era influenced by compensation: findings from a recent survey in Israel. Health Aff (Millwood). 2020:101377hlthaff202000382. 10.1377/hlthaff.2020.00382 32271627

[10] Machida M, Nakamura I, Saito R, Nakaya T, Hanibuchi T, Takamiya T (2020). Adoption of personal protective measures by ordinary citizens during the COVID-19 outbreak in Japan. Int J Infect Dis..

[11] Nonaka D, Morikawa H, Arioka H, Kobayashi J, Shoda R, Mizoue T (2011). Behavior of adult influenza patients during the 2009 pandemic after outpatient clinic presentations at a hospital in Tokyo. Japan. Trop Med Health..

[12] Blake KD, Blendon RJ, Viswanath K (2010). Employment and compliance with pandemic influenza mitigation recommendations. Emerg Infect Dis..

[13] Japanese Ministry of Health, Labour and Welfare. Basic policies for novel coronavirus disease control by the government of Japan (summary). 2020. https://www.mhlw.go.jp/content/10900000/000631465.pdf. Accessed 4 June 2020.

[14] GOV.UK. Our plan to rebuild: the UK Government’s COVID-19 recovery strategy. 2020. https://www.gov.uk/government/publications/our-plan-to-rebuild-the-uk-governments-covid-19-recovery-strategy/our-plan-to-rebuild-the-uk-governments-covid-19-recovery-strategy. Accessed 4 June 2020.

[15] Tang B, Bragazzi NL, Li Q, Tang S, Xiao Y, Wu J (2020). An updated estimation of the risk of transmission of the novel coronavirus (2019-nCov). Infect Dis Model..

[16] Japanese Society of Travel and Health. Novel coronavirus disease 2019 (COVID-19) infection prevention guideline for workplace. 2020. https://plaza.umin.ac.jp/jstah/pdf/corona01.pdf. Accessed 4 June 2020. (Only Japanese).

[17] U.S. Centers for Disease Control and Prevention. Discontinuation of isolation for persons with COVID-19 not in healthcare settings interim guidance. 2020. https://www.cdc.gov/coronavirus/2019-ncov/hcp/disposition-in-home-patients.html. Accessed 4 June 2020.

[18] Paulhus DL (1984). Two-component models of socially desirable responding. J Pers Soc Psychol..

[19] European Centre for Disease Prevention and Control. Isolate at home if sick. 2020. https://www.ecdc.europa.eu/sites/default/files/documents/Leaflet-Covid-19_Isolation-and-quarantine.pdf. Accessed 4 June 2020.

[20] Wright KB. Researching Internet-based populations: advantages and disadvantages of online survey research, online questionnaire authoring software packages, and web survey services. J Comput Mediat Commun. 2017;10(3).

[21] Statistics Bureau of Japan. 2015 Population Census. 2019. https://www.e-stat.go.jp/en/stat-search/files?page=1&toukei=00200521&tstat=000001080615. Accessed 4 June 2020.

[22] Peduzzi P, Concato J, Kemper E, Holford TR, Feinstein AR (1996). A simulation study of the number of events per variable in logistic regression analysis. J Clin Epidemiol.

[23] Japanese Ministry of Health, Labor and Welfare. Expert Meeting on the Novel Coronavirus Disease Control Analysis of the Response to the Novel Coronavirus (COVID-19) and Recommendations (Exerpt). 2020. https://www.mhlw.go.jp/content/10900000/000611515.pdf. Accessed 4 June 2020.

[24] Prime Minister of Japan and His Cabinet. Declaration of a State of Emergency in response to the Novel Coronavirus Disease. 2020. https://japan.kantei.go.jp/ongoingtopics/_00020.html. Accessed 4 June 2020.

[25] Coughlin SS (1990). Recall bias in epidemiologic studies. J Clin Epidemiol.

